# Prediction of the Mortality Risk in Peritoneal Dialysis Patients using Machine Learning Models: A Nation-wide Prospective Cohort in Korea

**DOI:** 10.1038/s41598-020-64184-0

**Published:** 2020-05-04

**Authors:** Junhyug Noh, Kyung Don Yoo, Wonho Bae, Jong Soo Lee, Kangil Kim, Jang-Hee Cho, Hajeong Lee, Dong Ki Kim, Chun Soo Lim, Shin-Wook Kang, Yong-Lim Kim, Yon Su Kim, Gunhee Kim, Jung Pyo Lee

**Affiliations:** 1 Department of Computer Science and Engineering, College of Engineering, Seoul National University, Seoul, South Korea; 2 Department of Internal Medicine, Ulsan University Hospital, University of Ulsan College of Medicine, Ulsan, South Korea; 3 College of Information and Computer Sciences, University of Massachusetts Amherst, Massachusetts, United States; 4 School of Electrical Engineering and Computer Science, Gwangju Institute of Science and Technology (GIST), Gwangju, South Korea; 5 Department of Internal Medicine, Kyungpook National University College of Medicine, Daegu, South Korea; 6 Department of Internal Medicine, Seoul National University Hospital, Seoul, South Korea; 7 Department of Internal Medicine Seoul National University College of Medicine, Seoul, South Korea; 8 Department of Internal Medicine, Seoul National University Boramae Medical Center, Seoul, South Korea; 9 Department of Internal Medicine, Yonsei University College of Medicine, Seoul, South Korea

**Keywords:** Outcomes research, Peritoneal dialysis

## Abstract

Herein, we aim to assess mortality risk prediction in peritoneal dialysis patients using machine-learning algorithms for proper prognosis prediction. A total of 1,730 peritoneal dialysis patients in the CRC for ESRD prospective cohort from 2008 to 2014 were enrolled in this study. Classification algorithms were used for prediction of N-year mortality including neural network. The survival hazard ratio was presented by machine-learning algorithms using survival statistics and was compared to conventional algorithms. A survival-tree algorithm presented the most accurate prediction model and outperformed a conventional method such as Cox regression (concordance index 0.769 vs 0.745). Among various survival decision-tree models, the modified Charlson Comorbidity index (mCCI) was selected as the best predictor of mortality. If peritoneal dialysis patients with high mCCI (>4) were aged ≥70.5 years old, the survival hazard ratio was predicted as 4.61 compared to the overall study population. Among the various algorithm using longitudinal data, the AUC value of logistic regression was augmented at 0.804. In addition, the deep neural network significantly improved performance to 0.841. We propose machine learning-based final model, mCCI and age were interrelated as notable risk factors for mortality in Korean peritoneal dialysis patients.

## Introduction

The prevalence of dialysis was calculated as 296 per million people (pmp), and that of renal replacement therapy (RRT) was assumed to be 709 pmp worldwide in 2010^1^; moreover, the incidence of end-stage renal disease (ESRD) is increasing steadily. Peritoneal dialysis (PD), a well-established RRT modality for patients with ESRD, varies greatly from country to country^2^. The prevalence of PD has been influenced by national policies of reimbursement, and there are differences in prevalence rates between countries^3^. Despite its clinical advantages, PD has been declining globally^3^, including in the Republic of Korea, where the proportion of PD decreased from 15% in 1990 to 7% in 2016^4^. PD versus hemodialysis (HD) has traditionally been of major benefit regarding residual renal function^5–7^, and recent studies showed that cognitive dysfunction improved relative to HD^8^. In addition, recent studies attempted remote monitoring with automated PD for home dialysis^9^. Even though it has many advantages, PD use has been limited in elderly debilitated patients and in those with comorbid diseases. To achieve the benefits of PD, it is necessary to accurately upgrade predictive risk factors for hard outcomes in PD patients.

Artificial intelligence algorithms in ESRD patients for predicting prognosis have been designed mainly to understand allograft outcomes in kidney transplant recipients^10–13^, because the immunologic and non-epidemiologic risks of renal transplant patients are complicated and affect prognosis^14^. Initially, attempts were made to predict early death in dialysis patients using a scoring system^15^. Subsequently, the number of studies for dialysis patients increased with the development of forecasting algorithms^16,17^. Most attempts were made to predict treatment responses to erythropoietin (EPO), an agent used to treat renal anemia in HD patients^18^, and these efforts were supported in multinational studies^19^. The reasons suggested were that EPO is relatively uniform in its capacity, treatment guidelines are clear-cut, and the artificial intelligence algorithm is easy to apply.

However, there is little use of artificial intelligence for diagnostic, therapeutic, and prognostic purposes in PD. In a recent report, three different machine-learning models, support vector machine (SVM), random forest (RF), and single-hidden-layer artificial neural network (ANN), were used to find immunologic biomarkers and report outperforming results^20^. Further, studies published in the Republic of Korea attempted to improve the discrimination index by using the modified Charlson-comorbidity index (mCCI) in incident PD patients^21^. In a later study, recalibration of mCCI was validated in incident PD patients and showed a different prognostic factor than in HD patients^22^. Therefore, in our study, we propose a novel prediction approach to PD patients’ survival, based on machine-learning techniques using nationwide prospective observational data for the augmentation of treatment strategies for PD patients in the Republic of Korea. In this study, we tried to demonstrate a novel data-driven approach using survival statistics to predict mortality.

## Results

### Baseline characteristics

**Figure 1:**
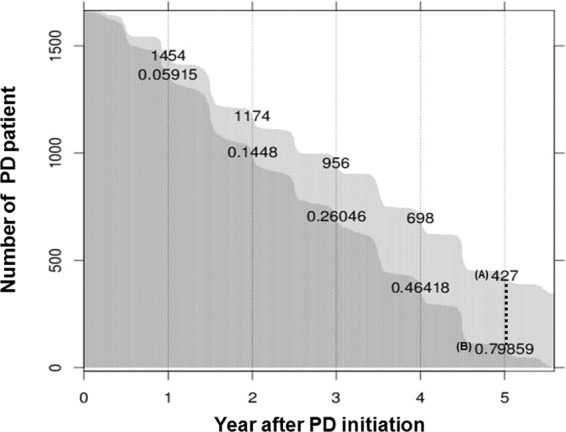
Patients’ follow up after peritoneal dialysis initiation. (**A**) Number of patients at the year of follow up at 1 year after peritoneal dialysis (PD) initiation (**B**) Ratio of non-survivor and survivor at 1 year after PD initiation.

Attributes used for modeling were presented according to mortality in PD patients (Table 1). Of the total 5223 dialysis patients, final analysis included 1,730 PD patients. A total of 343 patients (19.8%) died during the mean observation period of 30.18 ± 18.25 months (Fig. 1). The mean age was 62.3 ± 10.8 years for the non-survivor group and 50.3 ± 11.8 years for the survivor group (p < 0.001). Male patients in the non-survivor group were 63.3% (N = 217), and 55.2% of patients started dialysis due to diabetes (Table 1). Patients in the non-survivor versus survivor group had a significantly greater history of cardiovascular disease, diabetes, and increased mCCI scores. There were no differences between groups in body mass index (BMI), systolic blood pressure (SBP), and use of renin-angiotensin-aldosterone system blockade. Regarding laboratory findings at dialysis initiation, blood urea nitrogen (BUN), creatinine, phosphorus, and uric acid concentrations were all significantly lower in non-survivors than survivors (Table 1). Tables S1 and S2 presented the detailed protocol for missing data and follow-up patients’ data in online supplemental material.

**Table 1:** Baseline characteristics of study patients for peritoneal dialysis.

Variables	All (N = 1730)(%)	Non-survivor (N = 343)(%)	Survivor (N = 1387)(%)	P
Age (years)	52.7 ± 12.6	62.3 ± 10.8	50.3 ± 11.8	<0.001
Sex (male)	991 (57.3)	217 (63.3)	774 (55.8)	0.012
BMI (kg/m^2^)	23.3 ± 3.2	23.5 ± 3.2	23.2 ± 3.2	0.133
Primary renal disease				<0.001
Diabetes	617 (35.7)	179 (52.2)	438 (31.6)	
Hypertension	382 (22.1)	61 (17.8)	321 (23.1)	
Glomerulonephritis	292 (16.9)	30 (8.7)	262 (18.9)	
Cystic kidney disease	32 (1.8)	6 (1.7)	26 (1.9)	
Unknown	117 (6.8)	29 (8.5)	88 (6.3)	
Others	290 (16.8)	38 (11.1)	252 (18.2)	
History of CVD	461 (26.6)	149 (43.4)	312 (22.5)	<0.001
History of DM	712 (41.2)	209 (60.9)	503(36.3)	<0.001
Dialysis duration (months)	59.1 ± 46.3	59.9 ± 46.2	58.9 ± 46.3	0.727
Smoking history (%)	151 (8.7)	18 (5.2)	133 (9.6)	0.011
Modified CCI	4.4 ± 2.0	5.9 ± 2.0	4.0 ± 1.8	<0.001
Use of RAAS blockade	963 (55.7)	191 (55.7)	772 (55.7)	0.993
Systolic BP (mmHg)	133 ± 21	132 ± 21	134 ± 21	0.134
Diastolic BP (mmHg)	79 ± 22	76 ± 13	80 ± 23	0.005
**Laboratory findings at dialysis initiation**				
Hemoglobin (g/dL)	10.1 ± 3.4	10.3 ± 1.5	10.1 ± 3.7	0.344
BUN	64.5 ± 31.3	55.8 ± 28.7	66.5 ± 31.6	<0.001
Creatinine	8.9 ± 4.5	7.7 ± 3.9	9.1 ± 4.5	<0.001
Calcium	8.4 ± 0.9	8.5 ± 0.9	8.3 ± 1.0	0.070
Phosphorus	5.2 ± 1.6	4.7 ± 1.5	5.3 ± 1.6	<0.001
Uric acid	7.4 ± 2.0	7.0 ± 1.9	7.5 ± 2.0	<0.001
Total cholesterol	171 ± 44	168 ± 43	172 ± 44	0.195
Albumin	3.69 ± 2.19	3.67 ± 3.43	3.69 ± 1.79	0.908
Intact-PTH	290.6 ± 297.7	247.8 ± 282.1	300.8 ± 300.3	0.016
β2-microglobulin	51.2 ± 82.3	48.9 ± 70.6	51.8 ± 85.3	0.697

CVD, cardiovascular disease; DM, diabetes mellitus; MCCI, modified Charlson comorbidity index; RAAS, renin-angiotensin-aldosterone system.; SBP, systolic blood pressure; DBP, diastolic blood pressure; BUN, blood urea nitrogen; PTH, parathyroid hormone.

*Values are presented as n (%) for categoric variables, and mean ± standard deviation for continuous variables.

### Results of a conventional algorithm for predicting 5-year patients’ survival

**Figure 2:**
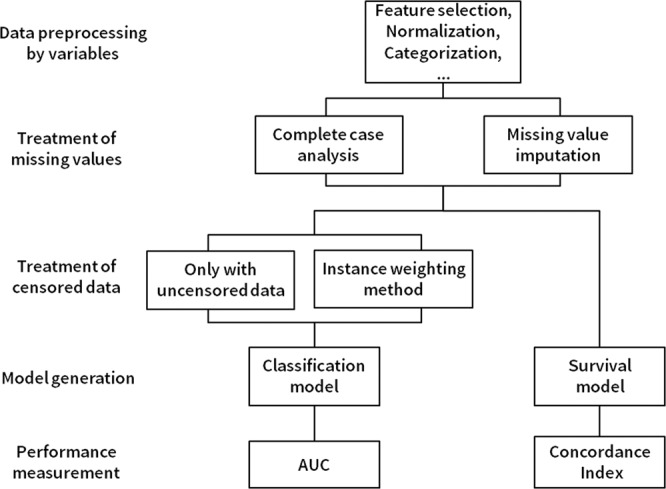
Model structure.

In this study, we presented the results chosen for model parameters, including imputation method, weighting methods, validation method and ratio, test-set size, training and test performance of the test set, and validation of the mixed dataset (Fig. 2). The performance of the machine-learning algorithm for classification is compared in Table 2, Table S3, and S4, according to test performance using the area under the curve (AUC) with different settings. These tables show predictive performances of the binary classification tree, derived from the dataset with the conventional multiple-learning method, for the expected probability of death after 5 years (%).

**Table 2:** Performance of the 5-year classification model without weighting methods in PD patients.

Validation method	Validation ratio	Test set size	Main algorithm	Hyperparameters^*^	Test performance
One validation	0.285	95	Bagging	nbagg = 10	0.6863
Cross-validation		95	Bagging	nbagg = 70	0.7407
**One validation**	**0.285**	**95**	**Decision tree**	**cp** = **-1/maxdepth** = **4**	**0.5782**
Cross-validation		95	Decision tree	cp = -1**/**maxdepth = 4	0.7222
One validation	0.285	95	Lasso	lambda = 2e-04	0.8105
Cross-validation		95	Lasso	lambda = 0.05	0.7979
		95	Logistic regression	Nothing	0.8219
One validation	0.285	95	Random forest	ntree = 800	0.7258
Cross-validation		95	Random forest	ntree = 1000	0.7535
One validation	0.285	95	Ridge	/lambda = 2e-04	0.8105
Cross-validation		95	Ridge	/lambda = 0.09	0.8164

Test ratio fix 0.3, and test performance were presented asAUC.

^*^We add explanation of the hyperparameters in the supplementary material.

**Figure 3:**
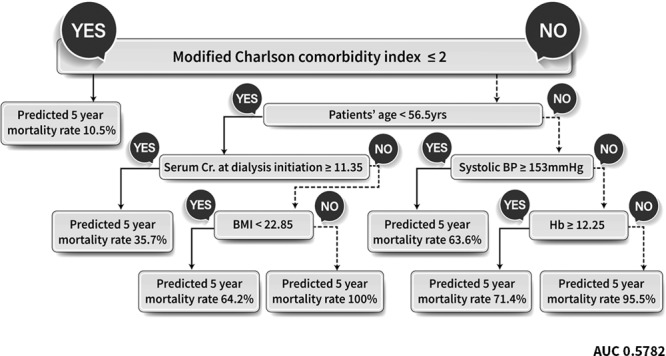
The 5-year mortality prediction after PD initiation using a decision tree (DT) model. The 5-year mortality of prediction rate is reported as a percentage (%). Decision tree for the training, test and validation data set, after stratified sampling, with ‘Y’ indicating a positive conclusion and ‘N’ a negative conclusion.

Figure 3 presents findings from the clinical decision model for the probability of death. From a range attribute of an existing decision-tree model, mCCI in the first node was the most important risk factor for mortality in PD patients. This revealed that the 5-year estimated mortality rate was only 10.5%, if mCCI was <2 and was used in preference to age at dialysis initiation and various other risk factors. Age at the start of PD was chosen as the next node. If PD was started at 56.5 years, the 5-year mortality rate was expected to be 37.5% if starting creatinine value was ≥11.35 mg/dL. If it was <11.35 mg/dL, BMI was determined to be the next node. If BMI was ≥ 22.85 kg/m2, the 5-year mortality rate was expected to be as high as 100% (Fig. 3). If age at the start of PD was >56.5 years, and if SBP at the start of dialysis was ≥153 mmHg, the expected 5-year mortality rate was 63.6%. For SBP < 153 mmHg, hemoglobin (Hb) value affected SBP. If Hb at the start of dialysis was <12.25 mg/dL, there was a high expected mortality risk of 95.5%.

### Results of Survival decision tree modeling for predicting patients’ survival hazard ratio

We applied additional machine-learning algorithms such as SDT, bagging, random forest, and ridge and lasso models. These models used survival analysis statistics instead of Gini indexes or entropy indexes in partitioning rules for existing tree algorithms. Table 3, and S5 show the final results for survival model parameters as concordance index (C-index). The survival tree algorithm performed better than the conventional survival modeling such as a Cox regression (0.769 vs. 0.745) (Table 3). The predicted death risk in the SDT model was a survival risk HR, compared to mean survival hazards for the entire patient population.

**Table 3:** Performance of the prediction models for mortality by survival statistics without imputation methods in PD patients.

Validation method	Validation ratio	Test set size	Main algorithm	Hyperparameters^*^	Test performance
Cross-validation		357	Survival tree	cp = 0.016**/**maxdepth = 6	0.7914
**One validation set**	**0.285**	**357**	**Survival tree**	**cp** = **0.014/maxdepth** = **6**	**0.7693**
Cross-validation		357	Survival ridge	**/**lambda = 0.07	0.7610
One validation set	0.285	357	Survival ridge	**/**lambda = 0.09	0.7593
Cross-validation		357	Survival random forest	splitrule = logrank**/**ntree = 1000	0.7479
One validation set	0.285	357	Survival random forest	splitrule = logrank**/**ntree = 800	0.7477
Cross-validation		357	Survival Lasso	lambda = 0.05	0.7599
One validation set	0.285	357	Survival Lasso	lambda = 0.07	0.7576
Cross-validation		357	Survival bagging	nbagg = 150	0.7631
One validation set	0.285	357	Survival bagging	nbagg = 10	0.7505
		357	Cox regression	Nothing	0.7458

Test ratio fix 0.3, and test performance were presented as concordance index. ^*^We add explanation of the hyperparameters in the supplementary material.

**Figure 4:**
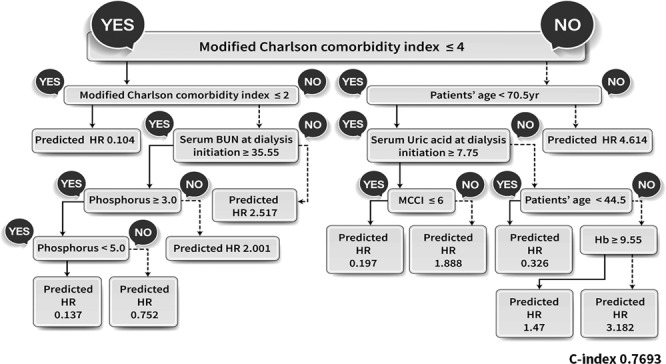
The patients’ survival prediction after PD initiation using survival hazard ratio modeling. The relative mortality risk is presented as a survival hazard ratio (HR). The survival decision tree for the training, test and validation data set, after stratified sampling, with ‘Y’ indicating a positive conclusion and ‘N’ a negative conclusion.

The most important factor in our additional analysis model was mCCI (Fig. 4). Companion comorbidities were divided into ≤4 and >4 (Fig. 4). Mortality was lowest, at HR 0.104, for all patients, regardless of other factors, in the mCCI ≤2 group. Conversely, there was a significant correlation with age from mCCI score ≥5 points. When mCCI score was ≥5 points and patients were aged >70.5 years of age, the HR for death was 4.614 times greater than that for overall mortality.

In patients with an mCCI score ≥5 but who were aged <70.5 years, uric acid level at the start of dialysis was the next decision node. If uric acid was >7.75 mg/dL, the survival HR was relatively low at 0.197 (for mCCI score of 5–6) but increased to 1.888 (for mCCI score ≥7.0). If uric acid levels in these patients were <7.75 mg/dL, the next node was age 44.5 years (Fig. 4). When age was <44.5 years and mCCI score was >5, there was a low mortality HR of 0.326, even when uric acid was <7.75 mg/dL at PD initiation. If patients were aged >44.5 years, the next node was Hb. If Hb was >9.55 g/dL, the HR of death was 1.47, but this increased to 3.182 if Hb was <9.55 g/dL (Fig. 4).

### Results of subgroup analysis for high risk patients

Next, additional analysis was performed in high risk subgroup using conventional Cox regression to find the effect of MCCI risk on each subgroup. We divide MCCI to groups according to the result of Figs. 3 and 4, and the risk group was collected into three groups, Low group MCCI 0–2; Moderate group 3–5; High group, MCCI score 6. Table 4 presented the results of Cox regression analysis. Multivariate analysis was performed with adjustment confounding, including such as age, sex, primary renal disease, smoking history, dialysis duration, BUN, systolic BP, BMI, Hb, calcium, history of DM, CVD, usage of RAAS blockade, and serum albumin. Table 4 showed that high MCCI group is an independent risk factor for PD patients after adjustment confoundings. Univariate analysis showed that high MCCI group have increased for the mortality risk in low albumin group and male gender. However, in old age group, there was no significant association of MCCI group and mortality risk.

**Table 4:** Cox regression analysis for the mortality according to the modified Charlson comorbidity index (mCCI) group in the high risk patients.

Subgroup	Model	Univariate			Multivariate		
		HR	95% CI	P	HR	**95% CI**	**P**
Overall group	Low	Ref.			Ref.		
	Moderate	6.135	(3.318, 11.342)	<0.001	2.700	(0.911, 7.997)	0.073
	High	17.708	(9.632, 32.557)	<0.001	4.618	(1.398, 15.256)	0.012
Male group	Low	Ref.			Ref.		
	Moderate	3.456	(1.742, 6.905)	<0.001	1.549	(0.509, 4.713)	0.441
	High	9.236	(4.691, 18.186)	<0.001	2.565	(0.735, 8.957)	0.140
Age ≥ 60 group	Low	Ref.			Ref.		
	Moderate	0.528	(0.073, 3.804)	0.526	0.417	(0.050, 3.496)	0.420
	High	0.988	(0.138, 7.068)	0.990	1.031	(0.116, 9.138)	0.978
History of DM group	Low	Ref.			Ref.		
	Moderate	1.506	(0.208, 10.888)	0.685	0.522	(0.067, 4.075)	0.535
	High	4.063	(0.568, 29.057)	0.162	0.757	(0.096, 5.998)	0.757
Low albumin group (≤3.5 g/dl)	Low	Ref.			Ref.		
	Moderate	2.792	(0.974, 8.004)	0.056	1.726	(0.468, 6.364)	0.412
	High	9.182	(3.342, 25.229)	<0.001	3.114	(0.744, 13.027)	0.120

mCCI group; Low, mCCI score 0–2; Moderate, mCCI score 3–5; High, mCCI score ≥6.

Multivariate analysis was done with adjustment confounding including such as age, sex, primary renal disease, smoking history, dialysis duration, BUN, systolic BP, BMI, Hb, Calcium, history of DM, CVD, usage of RAAS blockade, and serum albumin.

CVD, cardiovascular disease; DM, diabetes mellitus; RAAS, renin-angiotensin-aldosterone system.; SBP, systolic blood pressure; DBP, diastolic blood pressure; BUN, blood urea nitrogen; PTH, parathyroid hormone.

### Results of deep learning algorithm for predicting 5-year patients’ survival

We finally performed additional analysis to apply deep learning algorithm using longitudinal data to further enhance the prediction model. Mortality risk model was validated by the deep neural network algorithms, compare to conventional algorithms. Our proposed deep learning model is composed of Long Short-Term Memory networks (LSTM) and autoencoder. LSTM was introduced to deal with time-series data and autoencoder to deal with missing data.

**Figure 5:**
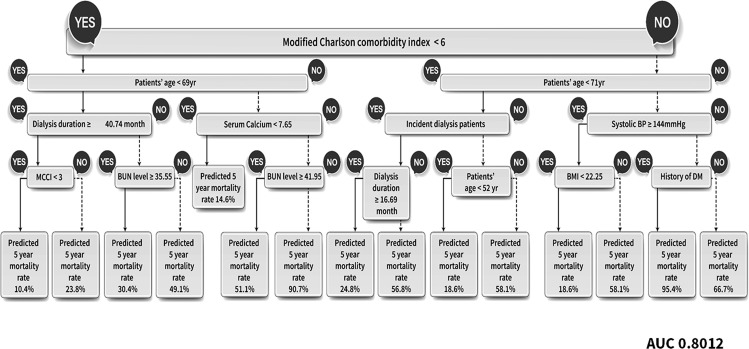
The 5-year mortality prediction after PD initiation using a decision tree (DT) model with repeated measured data. The repeated measured data include 24-hour urine volume, RAAS blockade use, and dialysis efficiency (weekly KT/V). The 5-year mortality of prediction rate is reported as a percentage (%). Decision tree for the training, test and validation data set, after stratified sampling, with ‘Y’ indicating a positive conclusion and ‘N’ a negative conclusion.

We analyze records of 1,127 prevalent and 603 incident PD patients, among which we use 26 independent attributes to learn this deep learning models including. The repeated measured data include 24-hour urine volume, RAAS blockade use, and dialysis efficiency (weekly KT/V). The study protocol of this study cohort was measured KT/V after 3 months of study enrollment, and the detailed protocol was presented in Table S2. Among the various conventional algorithms, the AUC value of logistic regression was the best at 0.804. Using these longitudinal data, the AUC of DT was also improved to 0.801 (Fig. 5). Our proposed deep learning model showed 0.840 when using only LSTM, and 0.858 when combined with an autoencoder (Table 5). Figure 5 presented the result of DT using longitudinal data (AUC 0.801). The most important factor in our additional analysis model was also found as mCCI (Fig. 5). Companion comorbidities were divided into ≤6 and >6 (Fig. 5), and age at the start of dialysis was the next decision node.

**Table 5:** Performance of the 5-year prediction model by deep neural network with autoencoder tree using repeated measured parameter.

Imputation method	Validation method	Validation ratio	Test set size	Main algorithm	Hyperparameters^*^	Test performance
**MICE/CART**	**One validation set**	**0.1**	**174**	**Decision Tree**	**cp** = **−1/maxdepth** = **4**	**0.8012**
MICE/CART			174	Logistic Regression	Nothing	0.8045
MICE/CART	One validation set	0.1	174	Ridge	**/**lambda = 0.02	0.8236
MICE/CART	One validation set	0.1	174	Lasso	lambda = 0.02	0.8193
MICE/CART	One validation set	0.1	174	Bagging	nbagg = 30	0.8332
MICE/CART	One validation set	0.1	174	Random Forest	ntree = 500	0.8381
MICE/CART	One validation set	0.1	174	Neural Networks	hunits = [8]	0.8066
Autoencoder	One validation set	0.1	174	Neural Networks	AE hunits = [16, 16]/FC hunits = [16]	0.8419
Autoencoder	One validation set	0.1	174	Long Short-Term Memory networks	LSTM hunits = 16/AE hunits = [16, 16]/FC hunits = [16]	0.8582

Test ratio fix 0.3, Training ratio fix 0.6, and test performance were presented as AUC.

^*^We add explanation of the hyperparameters in the supplementary material.

## Discussion

The purpose of this study was to accurately evaluate prognosis in PD patients using a nationwide prospective cohort and to select a machine-learning algorithm and neural network as novel tools. To the best of our knowledge, this is the first study to predict prognosis in PD patients using various machine-learning algorithms. The main conclusion of our study is that the presence and severity of comorbid diseases using mCCI is the first decision aid in determining future mortality risk in PD patients. Until now, studies attempting to predict prognosis using comorbidity index have been limited to HD patients and have lacked information about the predictive performance of specific score cutoffs and risk thresholds that could affect clinical applications^23^. According to the final conclusion of this study using survival statistics, the HR of death was only 0.104 for all patients with two or less comorbidities, regardless of age (Fig. 3). If there were two or less comorbidities at PD initiation, the predicted 5-year risk of death was only 10.5%, according to the decision-tree analysis (Fig. 2).

PD and HD each have advantages and disadvantages, and the survival rate in PD patients is comparable to that in HD patients in the Republic of Korea. However, there were inconsistent results in recent studies: for example, one report revealed better survival with PD than HD in the early period^24^, whereas another documented comparable mortality rates in PD and HD patients^25^. In elderly patients, there was no survival benefit for PD over HD in either study^24,25^. In the United States, there were at least 6,538 PD patients in 2007, and increased to 12,095 by 2016^26–28^; in the Republic of Korea, the corresponding at 2016 was 6,842 patients, only 7% of the total RRT population^4,29,30^. The rapid decline in PD patients in the Republic of Korea is probably due to the aging of patients with ESRD. PD is a patient-centered treatment, so it is difficult to attempt in elderly patients^31,32^ or those with a rapid decline in functional status^33^. Thus, updated prognostic tools are urgently needed and have not been tried in Korean patients. In particular, the absence of such prognostic tools has led to a reluctance of nephrologists to discuss post-dialysis prognosis with their patients^34,35^. Therefore, accurate prognostic tools are required, not only for PD patients, but also for patients with advanced chronic kidney disease and renal physicians, to facilitate shared-decision making.

In this study, we suggest that age itself is not the only important prognostic factor. We also suggest a cutoff in comorbidity index, related to age, which may help in selecting patients for PD. In a recent meta-analysis^23^, CCI was the best predictor of mortality, and CCI can be easily calculated in the clinical setting and provide meaningful information. CCI is a method of classifying patient complications based on ICD (International Classification of Diseases) diagnostic codes found in administrative data, such as hospital summary data^36^. Each complication category has associated weighting (from 1–6) based on the adjusted risk of death or resource use, and the sum of all weightings is each patient’s single complication score.

The CCI was originally developed in 1987. Charlson *et al*.^36^ identified 19 categories of associated diseases and weights for each category based on the adjusted 1-year relative risk of mortality. The sum of each weight is summed to yield a single equality score for each patient. However, this method has been criticized for applying CCI values directly to ESRD patients. Among the chronic disease indices validated in the dialysis population, CCI has been most widely used in statistical analyses due to its simplicity and ability to predict mortality; the original CCI has been validated in PD patients^37,38^. However, use of the original CCI has been questioned in patients with ESRD because of the proportion of complications within the CCI: there has been criticism that death in ESRD patients will not reflect the differences in effectiveness due to complications. In previous studies, a modification of CCI based on the proportion of relapsed complications, called mCCI, improved the predictability of mortality in dialysis populations. In these studies, complications such as cerebrovascular disease, congestive heart failure, diabetes, and myocardial infarction were reassigned to higher proportions of patients^38,39^. These patterns were observed in studies performed between 1999 and 2001 that applied mCCI in both HD and PD patients^39^. However, this study involved HD^39^, and studies involving only incident patients with PD in the Republic of Korea had a lower risk of comorbidities associated with cardiovascular disease^21^. This reflects the fact that PD patients have lower cardiovascular risk than HD patients.

One important meaning of our study is that machine-learning has increased usefulness of the mCCI, because the original CCI was designed to include only combinations identified from ICD-10 codes; however, there could have been clinical situations where it was necessary to consider all diagnoses of items not covered by the original CCI^36^. Because the original CCI was designed for use with very specific ICD coding found in hospital abstracts data, the medical conditions not covered in ICD coding could be overlooked. However, in our study cohort of ESRD patients, mCCI was calculated more accurately by the clinical research coordinator interviewing patients individually and then checking comorbidities. For example, if the cause of ESRD was diabetes, two points were allocated, as before, but if diabetes developed after ESRD, one point was allocated and two points were assigned for moderate or severe renal disease.

In the final SDT model (Fig. 4), the group with mCCI score >4 had the highest mortality among patients aged ≥70.5 years, as we expected (HR 4.614). Interestingly, uric acid level was determined as the next decision node in the group with mCCI score >4 points and aged <70.5 years. In this group, patients with the higher the concentration of uric acid at the start of dialysis (cutoff 7.75 mg/dL) showed the lower the risk of death (HR 0.197–1.888). In a previous study from the CRC for ESRD cohort, low uric acid level was associated with higher mortality in Korean dialysis patients, including PD and HD patients, after adjustment for nutritional markers, albumin, phosphorus, BMI, and subjective global assessment^40^. These results are consistent with our study findings. Intermediate-risk patients (mCCI score 3 or 4) were evaluated as the next decision node, according to serum BUN level. These patients had an increased risk of death (HR 2.517) if BUN level at the start of dialysis was low (cutoff 35.55 mg/dL). Taken together, our study results implicate that the mortality of PD patients might be related to nutritional and performance status at the dialysis initiation, irrespective of age itself.

In this process, we have not been satisfied with the performance of the model despite the analysis process, so we tried to strengthen the model by solving two problems after our CRC-ESRD cohort through deep learning algorithm i) Time-sequential longitudinal observational nature of data was attempted for overcome and performed deep learning algorism such as recurrent neural network (RNN) and Long Short Term Memory networks (LSTM) (ii) missing data was managed by Auto encoder (AE) were used to strengthen the model (Table 5). (i) The first feature of longitudinal observational cohort is the presence of time-variable attributes. The changing of those attributes might have played an important role in predicting the target variable. The recurrent neural networks (RNN) is a type of artificial neural network, and the connection between units has a cyclic structure^41^. These structures allow states to be stored inside the neural network to model time-variable dynamic attributes, while, unlike the conventional feed-forward artificial neural network, the RNN can process sequence-type inputs using internal memory. Thus, the RNN can process data with time-variable characteristics. In the case of vanilla RNN, gradients cannot be propagated normally, either vanished or exploded if the input sequence is long during the training process. This is called the problem of Long-Term Dependencies (LTD)^42^. To solve this problem, the special case of RNN, Long Short Term Memory networks (LSTM), was introduced. LSTM unit consists of an input gate, an output gate, a forget gate and a memory cell. The process is as follows in Figure S1. Figure S1 shows the structure of applying RNN/LSTM to the classification model, X is a static variable, and Xt is a time-dependent variable. In the study protocol of our cohort as Figure S1, time-dependent variables such as 24-hour urine volume, RAAS blockade use, and dialysis efficiency (weekly KT/V) were traced at 0/3/12 months (Table S2) and expressed the use of them as input values of RNN/LSTM units according to time order. (ii) The second feature of inevitable nature for observational cohort is the existence of various type of missing data. To solve these problems, we used Auto-Encoder (AE), which is a neural network that simply predicts the input value as an output value. If we set the number of nodes in the hidden layer to less than the input layer, AE can learn the compact representation of the input. This constraint enables us to learn how to express data efficiently, and it is possible to use this AE to express information including missing values as shown in Figure S2. In the training process, some input variable values are removed randomly, and AE is trained to restore them as the original values. In the inference process, the encoding value of the input is utilized regardless of the existence of the missing value.

Among the various algorithms using repeated measurement data, the AUC value of logistic regression was increased to  0.804. Using these longitudinal data, the AUC of DT was also improved to 0.801 (Fig. 5). Our proposed deep learning model showed 0.840 when using only LSTM, and 0.858 when combined with an autoencoder (Table 5). Finally, deep learning algorithm such as our proposed deep learning model with LSTM and autoencoder, especially repeated measured parameters, showed that the mortality risk of PD patients could be utilized more effectively.

Recently, the use of artificial intelligence in medical research has advanced rapidly^43^, and the feasibility of a machine-learning approach has increased, especially in diseases influenced by various prognostic factors, such as in ESRD, where application of a machine-learning approach can now be further expanded. The purpose of this study was to accurately predict prognosis in PD patients by focusing on comorbidities and clinical information at dialysis initiation. An elderly patient with high mCCI (≥5) on PD was associated with a 4.61-fold increase in the risk of death, while low mCCI patients (≤2) were associated with a 0.10 hazard ratio. We suggest information about the predictive performance of specific score cutoffs and risk thresholds that could be feasible for clinical PD applications. In conclusion, older adults are not unconditionally incapable of undergoing PD, and it may be helpful to calculate mCCI by accurately assessing comorbidities.

## Materials and Methods

### Data source and study patients

We conducted our analyses with data obtained from the database in the Clinical Research Center for ESRD (CRC for ESRD, NCT00931970), which is the only nationwide multicenter and prospective cohort of Korean ESRD. Data are collected from 36 general and teaching hospitals in the Republic of Korea. Our study included a total of 5,223 patients enrolled in the CRC for ESRD study, with 1,730 patients undergoing PD. All patients were aged ≥18 years and had received dialysis between August 2008 and December 2014. We have previously described the methods used to identify dialysis patients and their enrollment in the CRC for ESRD cohort^24,44,45^. Data were collected using a web-based platform (http://webdb.crc-esrd.or.kr) following the methods described in previous studies^24,44,45^. Out of 72 attributes in total, we analyzed 1,730 PD patient records using machine-learning algorithms with more than 50 attributes. Among these 50 attributes, we chose 23 independent attributes, which could affect all-cause mortality, to build our models. The attributes selected to build our models are specified in Table 1. The mCCI, which has been validated for dialysis patients^36^, was derived by reviewing patients’ medical histories and interviewing each patients by clinical research coordinator at enrollment.

### Problem statement and attributes used for modeling

In this study, we conducted a thorough analysis of different machine-learning algorithms to predict a survival rate at *N* years after PD initiation. In this section, we explain our data in detail and describe our models in Fig. 2. Finally, we provide comprehensive results of our experiments using different machine-learning methods. Outcome measurements for detecting mortality events, we had an approach to the mortality event and cause of death within 1 month after the event by the study protocol^24^ via clinical research coordinator (CRC), and re-assured by merging data from Statistics Korea with informed consents every end-of-year in study observation^46^. In our study patients’, a total of 156 patients were dropped out for kidney transplantation, and censored. When estimating the survival prediction at N years later, using data from people who have not been followed up after N years results in distortion. We tried to overcome the disadvantages with various methods. The previous study of our research had presented for detailed methods for using survival statics^13^. In brief, if the patient has already experienced an event (mortality) even though the follow-up period is short, it is used as a positive example. If the patient’s follow-up period is short, they are used both as positive and negative examples and give different weights to each, so-called instance weighting method (Fig. 2).

### Statistical analysis

In the present study, continuous and categorical variables were compared between the groups using the t-test and chi-square test, respectively. For the multivariate Cox model, we did not include MCCI as an adjusting covariate because of multicollinearity issues. We performed a stratified subgroup analysis using Cox regression model by age (>60 years), sex, and high risk groups (history of diabetes mellitus, low albumin level at dialysis initiation).

For extended methods related to the treatment of missing values, modeling process with data splitting, weighting method for classification, and detailed algorithm process including logistic regression, decision-tree, neural network, and deep learning algorithm process including recurrent neural network with autoencoder imputation, please refer to online supplemental data.

All of the statistical analyses were conducted using R statistical language (Version R 3.0.2, The Comprehensive R Archive Network: http://cran.r-project.org), and the MICE package was used as features for imputing missing values on continuous and categorical data. To implement the deep learning models, we use Python 3.6.5 and TensorFlow 1.14.0.

### Ethic statement

All patients were informed about the study, participated voluntarily, and provided written informed consent. Also, the institutional review board of each center approved the study. All investigators conducted this study in accordance with guidelines of the 2008 Declaration of Helsinki. Seoul National University Hospital Institutional Review Board approved the study (IRB number H-0905–047–281). The study was approved by the institutional review board at each center as follows [The Catholic University of Korea, Bucheon St. Mary’s Hospital; The Catholic University of Korea, Incheon St. Mary’s Hospital; The Catholic University of Korea, Seoul St. Mary’s Hospital; The Catholic University of Korea, St. Mary’s Hospital; The Catholic University of Korea, St. Vincent’s Hospital; The Catholic University of Korea, Uijeongbu St. Mary’s Hospital; Cheju Halla General Hospital; Chonbuk National University Hospital; Chonnam National University Hospital; Chung-Ang University Medical Center; Chungbuk National University Hospital; Chungnam National University Hospital; Dong-A University Medical Center; Ehwa Womens University Medical Center; Fatima Hospital, Daegu; Gachon University Gil Medical Center; Inje University Pusan Paik Hospital; Kyungpook National University Hospital; Kwandong University College of Medicine, Myongji Hospital; National Health Insurance Corporation Ilsan Hospital; National Medical Center; Pusan National University Hospital; Samsung Medical Center, Seoul; Seoul Metropolitan Government, Seoul National University, Boramae Medical Center; Seoul National University Hospital; Seoul National University, Bundang Hospital; Yeungnam University Medical Center; Yonsei University, Severance Hospital; Yonsei University, Gangnam Severance Hospital; Ulsan University Hospital; Wonju Christian Hospital (in alphabetical order)].

## Supplementary information

Supplemental material.
