# Supplementary material for: Prediction of the Mortality Risk in Peritoneal Dialysis Patients using Machine Learning Models: A Nation-wide Prospective Cohort in Korea
Source: Sci Rep. 2020 May 4;10:7470. doi: 10.1038/s41598-020-64184-0 (PMC7198502; doi:10.1038/s41598-020-64184-0)
Supplement: Supplementary file 1 — Supplemental material. [file 41598_2020_64184_MOESM1_ESM.pdf]

[Title page]

Prediction of the Mortality Risk in Peritoneal Dialysis Patients using Machine Learning Models: A Nation-wide Prospective Cohort in Korea

[1,*]Junhyug Noh, [2,*]Kyung Don Yoo, [3]Wonho Bae, [2]Jong Soo Lee, [4]Kangil Kim, [5]Jang-Hee Cho, [6]Hajeong Lee, [6,7]Dong Ki Kim, [7,8]Chun Soo Lim, [9]Shin-Wook Kang, [5]Yong-Lim Kim , [6,7]Yon Su Kim, [1,¶]Gunhee Kim,[7,8,¶]Jung Pyo Lee

[1]Department of Computer Science and Engineering, College of Engineering, Seoul National University, Seoul, South Korea

[2]Department of Internal Medicine, Ulsan University Hospital, University of Ulsan College of Medicine, Ulsan, South Korea

[3]College of Information and Computer Sciences, University of Massachusetts Amherst, Massachusetts, United States

[4]School of Electrical Engineering and Computer Science, Gwangju Institute of Science and Technology (GIST), Gwangju, South Korea

[5]Department of Internal Medicine, Kyungpook National University College of Medicine, Daegu, South Korea

[6]Department of Internal Medicine, Seoul National University Hospital, Seoul, South Korea

[7]Department of Internal Medicine Seoul National University College of Medicine, Seoul, South Korea

[8]Department of Internal Medicine, Seoul National University Boramae Medical Center, Seoul, South Korea

[9]Department of Internal Medicine, Yonsei University College of Medicine, Seoul, South Korea

*J Noh and KD Yoo contributed equally to the manuscript as lead authors.

The Table of Contents for Supplemental Material

Treatment of missing values

Table S1. The number of missed values in each observation

Modelling process with data splitting

Table S2. Longitudinal measurement for time-sequential information in the study cohort using deep neural network algorithm

Approach to classification problems using individual learners and ensemble variants

Table S3. Performance of the 5-year prediction model by conventional decision tree with imputation, and without weighting methods in PD patients

Table S4. Performance of the 5-year prediction model by conventional decision tree with weighting methods in PD patients

Weighting method for classification

Logistic regression

Decision-tree

Neural network

Approach to survival problems using individual learners and ensemble variants

Table S5. Performance of the prediction models for mortality by survival statistics with imputation methods in PD patients

Deep leaning algorithm process including recurrent neural network with autoencoder imputation

Figure S1. The longitudinal data management for the recurrent neural network (RNN) and long- and short-term memory network (LSTM)

Figure S2. The missing value learning (a) utilizing the auto encoder, (b) the inference process, (c) form combined with RNN

Treatment of missing values

Our data were collected over 7 years from 2008–2014, so it is inevitable to have some missing values. Of the total 1,730 observations, 38.5% contained at least one missing value. Table S1 shows numbers of observations for the given numbers of missing values. To manage these missing values, we used the following two methods: complete case analysis only, using existing attributes; and imputation with, in our case, Multivariate Imputation by Chained Equation (MICE). The MICE, defined in statistical libraries in R (version R 3.4.4; The Comprehensive R Archive Network: http://cran.r-project.org), creates imputations for multivariate missing values of both continuous and categoric data based on fully conditional specification, where each incomplete variable is imputed by a separate model, MICE method, with imputation of missing values for supplemented attributes [1]. Although several observed values were randomly drawn as the imputation, we drew only one value for each missing value.

Note that imputation methods were applied to observations with ≤4 missing values. That is, if an observation had too many missing values, imputation methods, which estimated missing values based on other existing attributes, could not properly impute missing values. If an observation contained >4 missing values, we simply dropped those values. Overall, we used 1,664 observations from a total of 1,730 patients (**Table S1**).

Table S1. The number of missed values in each observation

The number of missed values in each observation	0	1	2	3	4	5	6	7	8	9	10	11	12
Frequency	1063	226	251	81	43	13	9	4	1	1	1	22	14

Cumulative frequency	1063	1289	1540	1621	1664	1677	1686	1690	1691	1692	1693	1715	1729

Modelling process with data splitting

For experiments, we split our data into training (70%) and test (30%) sets. Due to the limited quantity of data, we performed a 5-fold cross-validation to prevent our model from being overfitted. After the cross-validation, we evaluated our model using the test sets. We set five different seeds to measure model performance using concordance index as a main criterion. We applied deep learning using longitudinal data. The repeated measured data include 24-hour urine volume, RAAS blockade use, and dialysis efficiency (weekly KT/V). The study protocol of this study cohort was measured KT/V after 3 months of study enrollment, and the detailed protocol was presented in **Table S2**.

Table S2. Longitudinal measurement for time-sequential information in the study cohort using deep neural network algorithm

	Information of RAAS blockade use	24 hr urine study as urine volume (ml)	Peritoneal Dialysis Adequacy as KT/V
Prevalence PD patient at 0 month	o	o	o
Incident PD patient at 0 month	o	o	x
Incident PD patient at 3 month	o	o	o
Prevalence PD patient at 12 month	o	o	o
Incident PD patient at 12 month	o	o	o

Approach to classification problems using individual learners and ensemble variants

We employed widely used, individual learning models (classification and regression trees, and logistic regression [2,3] and ensemble learning models [bagging and random forest] [1,4]). Our methods were detailed in a recently published study [5]. To predict survival at *N* years after PD initiation, we conducted various experiments using different algorithms. In this section, we introduce several classification algorithms and their ensemble variants. Besides the classification models, we also present machine-learning algorithms based on survival statistics. The performance of the machine-learning algorithm for classification is compared in Tables 2, Table S3, and S4, according to test performance using the area under the curve (AUC) with different settings.

Table S3. Performance of the 5-year prediction model by conventional decision tree with imputation, and without weighting methods in PD patients

Imputation method	Validation method	Validation ratio	Test set size	Main algorithm	Parameters	Training performance	Test performance
MICE/CART	One validation	0.285	129	Bagging	nbagg=50	1	0.7891
MICE/CART	Cross-validation		129	Bagging	nbagg=80	1	0.7233
MICE/CART	One validation	0.285	129	Decision tree	cp=-1 / maxdepth=2	0.8194	0.6869
MICE/CART	Cross-validation		129	Decision tree	cp=-1 / maxdepth=2	0.78	0.6135
MICE/CART	One validation	0.285	129	Lasso	lambda=0.003	0.8815	0.7988
MICE/CART	Cross-validation		129	Lasso	lambda=0.04	0.8566	0.7816
MICE/CART	One validation	0.285	129	Logistic regression	Nothing	0.889	0.8062
MICE/CART	One validation	0.285	129	Random forest	ntree=500	1	0.7783
MICE/CART	Cross-validation		129	Random forest	ntree=300	1	0.7426
MICE/CART	One validation	0.285	129	Ridge	lambda=0.002	0.8808	0.7815
MICE/CART	Cross-validation		129	Ridge	lambda=0.06	0.8749	0.8186

Test ratio fix 0.3, and test performance were presented as AUC.

MICE/CART, multivariate imputation by chained equation/classification and regression trees.

Table S4. Performance of the 5-year prediction model by conventional decision tree with weighting methods in PD patients

Imputation method	Validation method	Validation ratio	Test set size	Main algorithm	Parameters	Training performance	Test performance
nothing	One validation	0.285	95	Bagging	nbagg=130	0.6353	0.6615
nothing	Cross-validation		95	Bagging	nbagg=160	0.6351	0.7151
nothing	One validation	0.285	95	Decision tree	cp=-1 / maxdepth=2	0.56	0.6836
nothing	Cross-validation		95	Decision tree	cp=-1 / maxdepth=4	0.5693	0.6144
nothing	One validation	0.285	95	Lasso	lambda=0.03	0.5651	0.7767
nothing	Cross-validation		95	Lasso	lambda=0.02	0.5645	0.7761
nothing	One validation	0.285	95	Logistic regression	Nothing	0.5673	0.744
nothing	One validation	0.285	95	Random forest	ntree=700	0.6353	0.7609
nothing	Cross-validation		95	Random forest	ntree=1000	0.6351	0.75
nothing	One validation	0.285	95	Ridge	lambda=0.06	0.5662	0.7745
nothing	Cross-validation		95	Ridge	lambda=0.08	0.5645	0.7767
MICE/CART	One validation	0.285	129	Bagging	nbagg=190	0.6261	0.7063
MICE/CART	Cross-validation		129	Bagging	nbagg=170	0.6263	0.7272
MICE/CART	One validation	0.285	129	Decision tree	cp=-1 / maxdepth=2	0.5569	0.6837

MICE/CART	Cross-validation		129	Decision tree	cp=-1 / maxdepth=2	0.5584	0.6993
MICE/CART	One validation	0.285	129	Lasso	lambda=0.001	0.5675	0.7779
MICE/CART	Cross-validation		129	Lasso	lambda=0.004	0.5659	0.7595
MICE/CART	One validation	0.285	129	Logistic regression	Nothing	0.5665	0.7532
MICE/CART	One validation	0.285	129	Random forest	ntree=1000	0.6261	0.762
MICE/CART	Cross-validation		129	Random forest	ntree=1000	0.6263	0.7267
MICE/CART	One validation	0.285	129	Ridge	lambda=0.02	0.5638	0.7617
MICE/CART	Cross-validation		129	Ridge	lambda=0.04	0.5662	0.7645

Test ratio fix 0.3, and test performance were presented as AUC.

MICE/CART, multivariate imputation by chained equation/classification and regression trees.

Weighting methods were applied according to the methods of Zupan, *et al*.

Weighting method for classification

It was necessary to determine period to characterize the survival analysis problem as a classification problem. We set the period to 5 years. Thus, we redefined our problem as "whether a patient survives 5 years after PD initiation". This definition produced right-censored data [5], which were handled by either dropping, or applying the weighted method proposed by Zupan *et al.* [6]. The weighting method created two copies, 0 and 1, for each piece of right-censored data and assigned a probability for each case as a weight, based on survival function.

Logistic regression

One of the most common machine-learning algorithms is logistic regression. It is a generalized linear model (GLM) used for classification problems. Instead of assuming that a dependent variable is a normal distribution in the case of a linear regression model, it assumes that a dependent variable is a Bernoulli distribution. Hence, logistic regression converts a linear combination of independent variables to binary-valued outcomes using a logit function formulated as $\pi(\mathrm{X}) = 1/(1 + \exp(-\beta\mathrm{X}))$, where $\pi(\mathrm{X})$ indicates probability of the dependent variable, y, being in class 1 given the independent variables, or simply $p(y=1|X)$ [7]. A logistic regression model is trained to minimize a predefined cost function which, in our case, was defined as $cost(\hat{y}, y) = \sum(-y\log\hat{y} - (1-y)\log(1-\hat{y})$ where $\hat{y}$ is equivalent to $p(y=1|X)$. Further, to avert a problem of overfitting, which prevents a model from generalizing unseen data, we also applied Lasso and Ridge, which constrains the cost function using $\|w\|_1$ and $\|w\|_2^2$, respectively, so that it prevents a model being overfitted.

Decision-tree

The decision-tree algorithm, another commonly used classification algorithm, is a simple and intuitive yet robust machine-learning algorithm. It is easier to implement a decision-tree algorithm, and interpret its results, than many other machine-learning methods. Further, it is robust due to its nature of non-linearity [4]. We employed a classification and regression tree (CART) algorithm, which is a specific type of decision-

tree algorithm. CART forms a binary tree and gradually expands its leaf nodes to maximize purity measurement or equivalently minimize impurity measurement. Among three commonly used impurity measurements, we chose Gini index, which measures the impurity of internal nodes. The algorithm expands until it meets stopping rules specified as hyperparameters [4].

To enhance the performance of individual algorithms, ensemble methods are often employed. These methods are machine-learning algorithms that combine multiple base learners with the aim of improving predictive performance of the given base model. In this paper, we used bootstrap aggregating, also known as bagging [2], and random forest [3] as ensemble methods. Bagging consists of multiple base models independently trained on bootstrapped samples of the same size from the training dataset. In inference time, it aggregates output predictions by averaging and voting for regression and classification, respectively. The random forest algorithm adds more randomness to bagging. It not only bootstraps samples but randomly chooses a fixed number of attributes among all the attributes available and finds the best split using them [1]. In this way, it improves accuracy of the output predictions. We chose CART as a base learner for both bagging and random forest [4].

Neural network

A neural network is a network of neurons that aims to recognize underlying relationships of data through a process that imitates the way a human brain operates. It consists of input, hidden, and output layers. An input layer corresponds to the variables of input data. After a neural network takes input data through an input layer, it is passed into a hidden layer, which linearly combines the input data and modifies it using a nonlinear function, also known as an activation function. Then, output of the hidden layer is passed into either the next hidden layer or output layer. A neural network can approximate a function for both classification and regression problems. In our case, it was designed to solve the binary classification problem.

In general, a neural network can be formulated in a mathematical form as follows:

$$z_m = \sigma(\alpha_{0m} + {\alpha_m}^T x) \ , m = 1,2,\ldots,M$$

$$t_k = \beta_{0k} + {\beta_k}^T z, k = 1,2,\ldots,K$$

$$f_k(x) = g_k(t)$$

where $z = (z_1, z_2, \ldots, z_M)^T$ is a hidden layer and $t = (t_1, t_2, \ldots, t_M)^T$ is an output layer. Also, $\sigma(\cdot)$ and $g(\cdot)$ are activation functions, which add nonlinearity to the inputs.

The network can be trained by minimizing a loss function as a proxy to improve its performance, which in our case, is classification accuracy. Although there are many options for a loss function, in a classification task, cross entropy loss (defined below) is generally used. Due to nonconvexity of cross entropy loss, it is not possible to compute a global minimizer using analytical optimization methods. Instead, numerical optimization methods, such as gradient descent or its variants, are used to estimate a global minimizer.

Approach to survival problems using individual learners and ensemble variants

As a characteristic of observational cohort datasets, much data is censored. It is often omitted for the sake of simplicity, but this degrades the performance of a model due to insufficient follow-up. An alternative solution is to treat censored data as non-recurring samples (classification) and their follow-up times as survival times (regression).

Both of these solutions, however, introduce bias that is amplified when the rate of event occurrence is low. To avoid such bias and include all censored data, we modeled a Survival Decision Tree (SDT) algorithm using survival statistics [5,8].

As described in the previous subsection, a general decision-tree algorithm recursively finds the best attribute to split a node using an impurity measurement such as Gini index or entropy index, which measures impurity in a classified outcome. Conversely, SDT uses survival statistics as a split criterion. It expands its nodes to maximize the improvement, which is formulated as:

1) c_i: the observed event count for observation i; 2) t_i: the observation time for observation i; 3) observed event rate: $\hat{\lambda} = \frac{\#\, events}{total\ time} = \frac{\sum c_i}{\sum t_i}$; 4) within-node deviance: $D = \frac{1}{N}\sum\left[c_i \log\left(\frac{c_i}{\hat{\lambda}t_i}\right) - \left(c_i - \hat{\lambda}t_i\right)\right]$; and 5) maximize the improvement of: $D_{parent} - (D_{left} + D_{right})$.

As with a general decision-tree method, SDT expands until it meets stopping rules. For our experiments, we set the model to stop splitting when either split did not improve the fit by a certain threshold or the depth of any node reached a certain threshold. Through the stopping rule, we prevented the model from being overfitted to the training dataset. To boost performance of the STD model, we applied ensemble methods in a similar manner to the classification models. We employed both bagging and random forest with STD as a base model [8]. **Table 3, and S5** show the final results for survival model parameters as concordance index (C-index).

Table S5. Performance of the prediction models for mortality by survival statistics with imputation methods in PD patients

Validation method	Validation ratio	Test set size	Main algorithm	Parameters	Training performance	Test performance
One validation set	0.285	502	Survival tree	cp=0.016 / maxdepth=4	0.7547	0.7526
Cross-validation		502	Survival tree	cp=0.018 / maxdepth=4	0.7617	0.7526
Cross-validation		502	Survival ridge	lambda=0.02	0.7776	0.7353
One validation set	0.285	502	Survival ridge	lambda=0.1	0.7735	0.734
Cross-validation		502	Survival random forest	splitrule=logrank / ntree=100	0.9706	0.7236
One validation set	0.285	502	Survival random forest	splitrule=logrank / ntree=100	0.9715	0.716
One validation set	0.285	502	Survival Lasso	lambda=0.02	0.7698	0.7365
Cross-validation		502	Survival Lasso	lambda=0.01	0.7795	0.7333
Cross-validation		502	Survival bagging	nbagg=140	0.8286	0.7304
One validation set	0.285	502	Survival bagging	nbagg=30	0.8622	0.7269
One validation set		502	Cox regression	Nothing	0.7832	0.7205

Test ratio fix 0.3, and test performance were presented as concordance index.

Deep leaning algorithm process including a recurrent neural network with an autoencoder imputation

We have not been satisfied with the performance of the model despite its analysis process, so we tried to strengthen the model by solving two problems after our CRC-ESRD cohort by using a deep learning algorithm: i) The time-sequential longitudinal observational nature of data was attempted to overcome and perform deep learning algorithms, such as the recurrent neural network (RNN) and long short-term memory network (LSTM); (ii) missing data was managed by an autoencoder (AE), which was used to strengthen the model (**Table 5**).

(i) The first feature of the longitudinal observational cohort is the presence of time-variable attributes. Changes in these attributes might have played an important role in predicting the target variable. The recurrent neural network (RNN) is a type of artificial neural network, and the connection between its units has a cyclic structure.[9] These structures allow states to be stored inside the neural network to model time-variable dynamic attributes. Unlike conventional feed-forward artificial neural networks, the RNN can process sequence-type inputs using internal memory. Thus, the RNN can process data with time-variable characteristics. In the case of vanilla RNN, gradients cannot be propagated normally as they either vanish or explode if the input sequence is long during the training process. This is called the problem of long-term dependencies (LTD)[10]. To solve this problem, a special case of RNN, the LSTM, was introduced. An LSTM unit consists of an input gate, an output gate, a forget gate, and a memory cell. The process is shown in Figure S1. Figure S1 shows the structure when applying RNN/LSTM to the classification model; X is a static variable, and Xt is a time-dependent variable. In the study protocol for our cohort as shown in Figure S1, the time-dependent variables were traced at 0/3/12 months (Table S2) and their use was expressed as input values of RNN/LSTM units according to the time order. In the case of a patient without a tracking value, the first unit predicted the value of the target variable immediately ($P(\text{Death}|X, X_0)$ in Figure S1). In the case of the patient with a tracking value, the unit made predictions according to these changes ($P(\text{Death}|X, X_0, X_3)$ or $P(\text{Death}|X, X_0, X_3, X_{12})$).

Figure S1. The longitudinal data management for the RNN and LSTM.

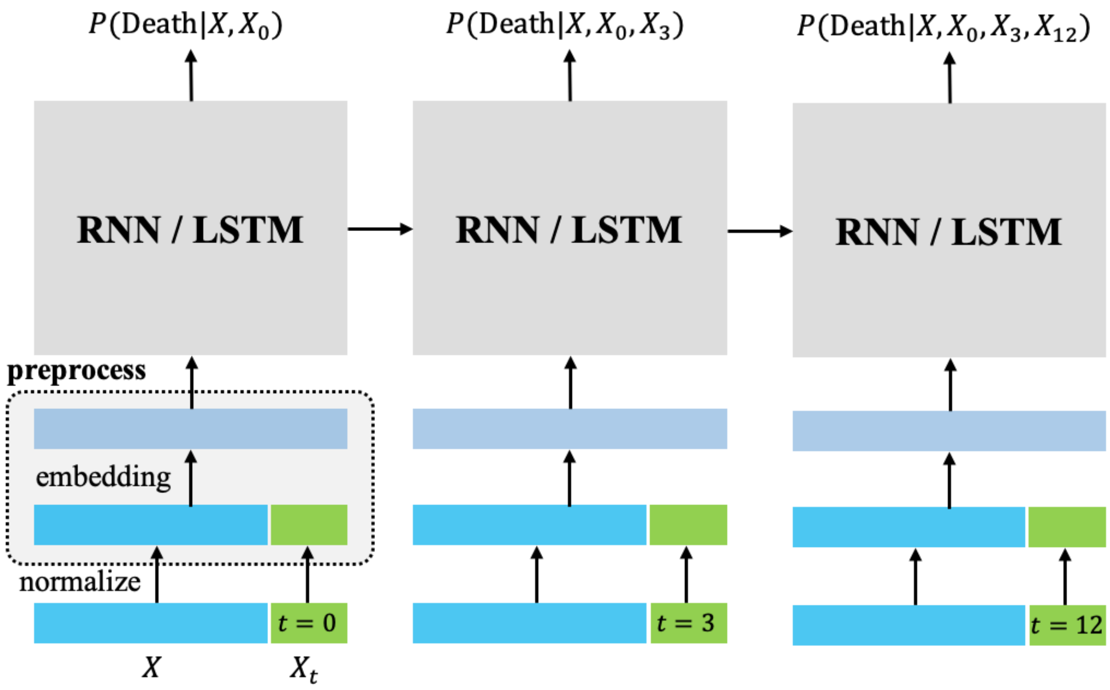

(ii) The second feature of inevitable nature for the observational cohort is the existence of missing data. When the data is missing values, the simplest processing method is a complete data analysis that omits the missing data. However, this method can cause two major problems. The first is that statistical significance can be lost due to a decrease in the size of the data, and the second is that the bias of the model as a result of the difference in the population distribution can occur. To solve these problems, we used an autoencoder (AE), which is a neural network that simply predicts the input value as an output value. If we set the number of nodes in the hidden layer to less than the input layer, the AE can learn the compact representation of the input. This constraint enables us to learn how to express data efficiently, and it is possible to use this AE to express information, including missing values, as shown in Figure S2. In the training process, some input variable values were randomly removed, and the AE was trained to restore them as the original values. In the inference process, the encoding value of the input was utilized regardless of the existence of the missing value. Figure S2(c) shows the overall structure in which the AE is combined with RNN/LSTM.

Among the various algorithms, the AUC value for logistic regression was the best at 0.804. Using these longitudinal data, the AUC of DT was also improved to 0.801 (**Figure 5**). Our proposed deep learning model was 0.840 when using only LSTM and 0.858 when combined with an autoencoder (**Table 5**).

Figure S2. The missing value learning (a) utilizing the AE, (b) the inference process, (c) form combined with RNN/LSTM.

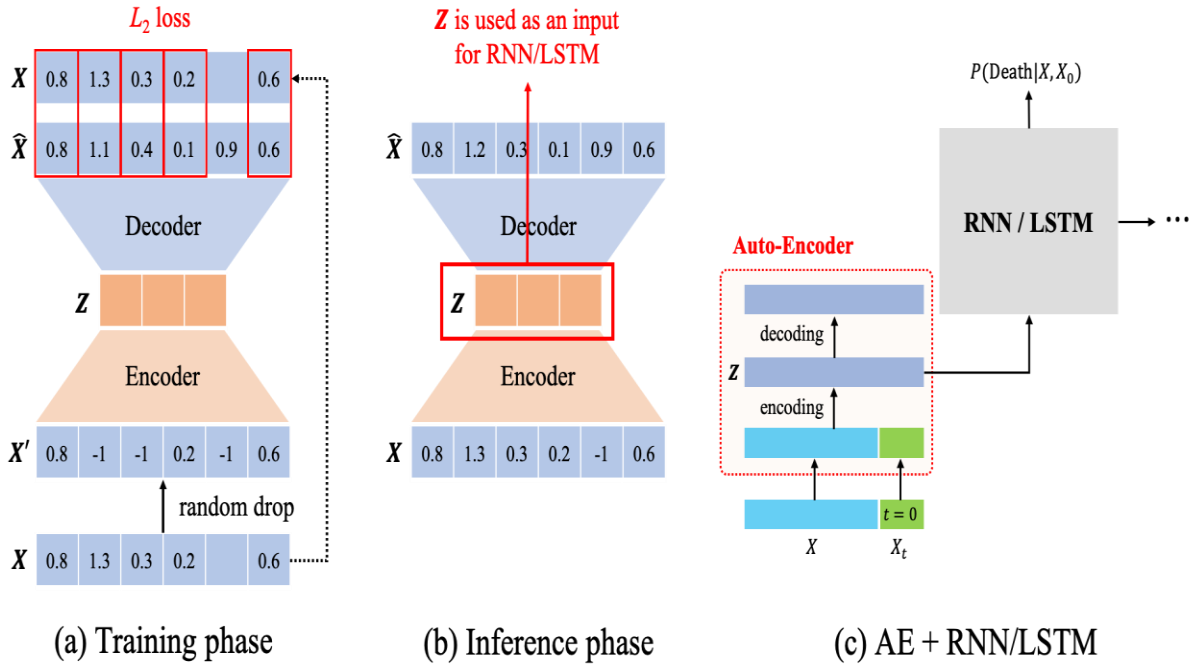

Hyperparameter Description

The hyperparameters for each algorithm in Tables 2, 3 and 5 of the main paper are as follows;

- Decision tree
  - cp: complexity parameter
  - maxdepth: maximum depth of any node of the final tree
- Bagging
  - nbagg: number of bootstrap replications
- Random forest
  - ntree: number of trees in the forest
  - splitrule: splitting rule
- Ridge, Lasso
  - lambda: weight of the penalty
- Neural networks
  - FC hunits: number of hidden units in FC layers
  - AE hunits: number of hidden units in Auto-Encoder layers
  - RNN hunits: numbr of hidden units in RNN cell

REFERENCE

1. Buuren, S., & Groothuis-Oudshoorn, K. Mice: Multivariate imputation by chained equations in R. *JSS.* **45**(2011).
2. Breiman, L. Bagging Predictors. *Mach. Learn* **24**, 123-140 (1996).
3. Breiman, L. Random forests. *Mach. Learn* (2001).
4. Breiman, L., Friedman, J., Stone, C.J., & Olshen, R.A. Classification and Regression Trees. *CRC press* (1984).
5. Yoo, K.D*., et al.* A Machine Learning Approach Using Survival Statistics to Predict Graft Survival in Kidney Transplant Recipients: A Multicenter Cohort Study. *Sci Rep* **7**, 8904 (2017).
6. Zupan B, D.J., Kattan MW, Beck JR, Bratko I. Machine learning for survival analysis: a case study on recurrence of prostate cancer. *Artificial Intelligence in Medicine* **20**, 59-75 (2000).
7. Dobson, A.J. An introduction to generalized linear models. *Journal of Statistical Planning and Inference* **32**, 418-420 (1992).
8. LeBlanc, M., & Crowley, J. Relative Risk Trees for Censored Survival Data. *Biometrics* 411-425 (1992).
9. Mikolov, T., et al. Recurrent neural network based language model. *International speech communication association* (2010).
10. Hochreiter, S., and Jürgen Schmidhuber. Long short-term memory *Neural computation* 1735-1780 (1997).
